# Production, Characterization, and Function of Pseudoislets from Perinatal Canine Pancreas

**DOI:** 10.1177/0963689719869004

**Published:** 2019-08-26

**Authors:** P. Czernichow, K. Reynaud, J. Kerr-Conte, E. Furthner, P. Ravassard

**Affiliations:** 1 Animal Cell Therapy, University Pierre et Marie Curie, Paris, France; 2 Ecole Nationale Vétérinaire d’Alfort, Maisons-Alfort, France; 3 PRC, UMR INRA0085, CNRS 7247, Centre INRA Val de Loire, Nouzilly, France; 4 University Lille, Inserm, CHU Lille, U1190 Translational Research for Diabetes, European Genomic Institute for Diabetes, EGID, Lille, France; 5 Institut du cerveau et de la moelle (ICM), Hôpital Pitié-Salpêtrière, Sorbonne Universités, UPMC Univ Paris 06, Inserm, CNRS, Paris, France

**Keywords:** pseudoislets, β cells, dog models, islet transplantation, diabetes

## Abstract

We evaluated the cell composition and function of canine pancreatic pseudoislets (PIs) produced from 42- to 55-day-old fetuses, 1- to 21-day-old pups, and an adult dog pancreas. After mild collagenase treatment, partially digested tissues were cultured for 2–3 weeks. PI production started on culture day 3, was marked for 6 to 9 days, and then stopped. PI production was greatest with the neonatal specimens, reaching about 12 million aggregates per litter (55-day-old fetus) or per pancreas (1-day-old pup). Cell composition at all stages was similar to that in adult pancreatic islets, with predominant β cells, scant α cells and, most importantly, presence of δ cells. Among pancreatic markers assessed by quantitative real-time PCR (qRT-PCR) mRNA assay, insulin showed the highest expression levels in PIs from newborn and adult pancreas, although these were more than 1000 times lower than in adult islets. Pdx1 mRNA expression was high in PIs from 55-day-old pancreases and was lower at later stages. Consistent with the qRT-PCR results, the insulin content was far lower than reported in adult dog pancreatic islets. However, insulin release by PIs from 1-day-old pups was demonstrated and was stimulated by a high-glucose medium. PIs were transplanted into euglycemic and diabetic SCID mice. In euglycemic animals, the transplant cell composition underwent maturation and transplants were still viable after 6 months. In diabetic mice, the PI transplants produced insulin and partially controlled the hyperglycemia. These data indicate that PIs can be produced ex vivo from canine fetal or postnatal pancreases. Although functional PIs can be obtained, the production yield is most likely insufficient to meet the requirements for diabetic dog transplantation without further innovation in cell culture amplification.

## Introduction

Almost 40 years ago, biologists in Uppsala, Sweden, described an innovative method for the large-scale isolation of islet-like structures, composed predominantly of beta cells, from cultured fetal rat pancreases previously subjected to mild collagenase digestion^1^. The material thus obtained, the exact origin of which remains unclear, has been designated by a variety of terms including “neonatal islets,” “islet-cell clusters,” and “pseudoislets” (PIs), which is the term used herein. The same method was subsequently used to isolate PIs from human fetal pancreases^2^. PIs can grow both in vitro and in vivo. Thus, in nude mice, transplanted PIs survived and grew for 2 months^3^. PIs from both rat and human pancreases exhibited only a weak insulin secretory response to glucose. Nonetheless, researchers showed strong interest in the production of fetal PIs, applying the Swedish method to other species. Viable PIs obtained by culturing collagenase-digested fetal porcine pancreases were shown to normalize blood glucose levels in nude mice within 2 months after transplantation^4^. The large-scale isolation of neonatal porcine PIs was also described^5,6^. Attempts to isolate PIs from fetal sheep were less successful, with a weak insulin response and poor growth after transplantation into nude mice^7,8^.

The objective of these studies was to find an abundant source of islets for transplantation in humans, an approach known as beta-cell therapy, which holds considerable promise for treating diabetes in humans^9^. The incidence of diabetes mellitus (DM) in dogs is similar to that in humans and has increased recently^10,11^. DM is caused by selective beta-cell destruction within the pancreatic islets^12^, whose mechanisms remain unknown. Having an abundant source of canine beta cells would not only allow transplantation as a treatment for diabetes, but also provide a model for investigating beta-cell transplantation with the goal of eventually developing this technique as a treatment for humans^13^.

Here, our objective was to investigate the feasibility of PI production in dogs using the method previously described in rats, pigs, sheep, and humans. More specifically, we aimed to identify the pancreatic development stage at which PI production was most effective and to determine whether the PIs were functional. Should production of functional PIs in large numbers be achieved, then PI production might constitute a source of beta cells for replacement therapy in diabetic dogs. In addition, the dog PI model should prove valuable for investigating the technical aspects of beta-cell transplantation in humans.

## Material and Methods

All procedures involving animals were submitted to and approved by the institutional review board of the Maisons-Alfort Veterinary School, Maisons-Alfort, France. Our methods complied with international regulations for the use of animals in experimental studies.

### Sources of Canine Pancreatic Tissue and Sample Collection Procedure

We obtained antenatal dog pancreas specimens at three developmental stages, namely, fetal day (F) 42 (three bitches, 18 fetal pancreases), F45 (two bitches, 13 fetal pancreases), and F55 (three bitches, 17 fetal pancreases), for a total of 48 pancreases. All antenatal samples were from a strain of beagle dogs raised at the Maisons-Alfort Veterinary School. Specimens were obtained by elective caesarean section. Fetal age was determined based on the time of the plasma progesterone surge indicating ovulation. Postnatal pancreases were obtained from four beagle dogs, including two that died on postnatal day 1 (D 1) (*n* = 2) of unknown causes and two that were euthanized due to recurrent seizures at the end of the third postnatal week (W3). Finally, a pancreas was obtained from an adult American Staffordshire terrier that was euthanized for legal reasons.

### Tissue Culture and PI Production

Each pancreas was dissected aseptically and placed in sterile ice-cold Hanks balanced salt solution (HBSS) supplemented with 2% bovine serum albumin, glucose (5.6 mM), and antibiotics (1% penicillin and 1% streptomycin). For the prenatal specimens, about 4–6 pancreases were obtained depending on developmental stage. Dissection was completed under a binocular microscope and the pancreas was cleared of adjacent tissue. The dissected pancreas was minced into fragments measuring about 1 mm^3^, which were washed twice in ice-cold HBSS then incubated at 37° in HBSS containing 6 mg/mL of collagenase A (Roche, Basel, Switzerland). After 6–8 min, the collagenase digestion was stopped by adding 20 mL of ice-cold HBSS. The fragments were washed twice and centrifuged, and the pellet was resuspended in 100 mL of HBSS. The suspension was stirred using a magnetic stirrer at room temperature to promote disaggregation of the tissue fragments. After 60 min, the suspension was centrifuged and the pellet was resuspended in ice-cold HBSS. After 2 washings, 9 mL of culture medium (RPMI 1640 with 10% fetal calf serum, 1% penicillin, and 1% streptomycin) was added to the pellet and the resulting suspension was plated on a B10 culture dish, which was maintained at 37°C in a humidified mixture of 95% air and 5% CO_2_. For experiments on F42 specimens, 6–7 pancreases were usually obtained, and the pellet resulting from 3–4 pancreases was plated on a single B10 culture dish. For F55 and later stages, the pellet from a single pancreas was plated on one or more B10 culture dishes depending on the size of the gland.

The RPMI culture medium was changed every 3 days. After 6–14 days of culture, the PIs were harvested either by gently blowing culture medium over the culture dish through the tip of a pipette or by collecting free-floating PIs from the culture dish supernatant. PI-containing medium was then centrifuged and the pellet collected, resuspended in the same buffer and stored in the incubator for further experiments.

### Preparation of Adult Islets of Langerhans

Islets of Langerhans were isolated from an adult dog pancreas by J Kerr-Conte at the university hospital in Lille, France. The pancreas was dissected and prepared for islet isolation after Wirsung canal catheterization and collagenase digestion, as described previously^14^. Before tissue processing, a fragment was harvested for PI production, as described above, and for immunohistochemistry studies.

### Preparation of Canine Tissue for Immunohistochemistry Studies

#### Pancreas

Immediately after surgery, a piece of each pancreas was dissected and fixed in 3.7% formaldehyde then embedded in paraffin. The pancreases from the 1-day-old, 3-week-old, and adult animals were obtained within 1 h after death and fixed in phosphate-buffered saline/10% formaldehyde prior to paraffin embedding, as previously described^15^.

#### PIs and Adult Islets of Langerhans

PIs were harvested after 6–12 days of culturing. PI-containing medium was centrifuged and after washing with HBSS, samples were fixed in phosphate-buffered saline/10% formaldehyde and then embedded in paraffin. Islets of Langerhans prepared from the adult dog pancreas were prepared for immunohistochemistry using the same procedure.

### Immunohistochemistry

Paraffin sections of prenatal and postnatal specimens were 4 µm and 5 µm, respectively. The sections were stained with guinea pig anti-insulin antibody (1/500; A0564, DakoCytomation, Carpinteria, CA, USA), rabbit anti-glucagon antibody (1/1000; 20076-Immuno, Euromedex, Souffelweyersheim, France), and rabbit anti-somatostatin antibody 1/500, DakoCytomation). The secondary antibodies were fluorescein Texas Red anti-guinea pig antibody (1/2000; 706-076-148) and anti-rabbit antibody (1/200; 711-096-152) (both from Jackson ImmunoResearch Labs, West Grove, PA, USA). Nuclei were stained with Hoechst 33342 fluorescent stain (1/5000; 62249, Thermo Fisher Scientific, Waltham, MA, USA).

For each specimen, a pool of 150–500 sections was obtained. The sections to be examined were taken at regular intervals from this pool and considered to be representative of the entire gland. Digital images were taken using an Axio Scan Z1 camera (Zeiss, Oberkochen, Germany) or an Olympus FluoView FV1000 confocal microscope (Olympus, Shinjuko, Tokyo, Japan).

### RNA Isolation and Quantitative Real-Time PCR (qRT-PCR) Procedure

Total RNA was isolated from the samples using the RNeasy Micro Kit 50 (Qiagen, Hilden, Germany; ref: 74004) according to the manufacturer’s instructions. First-strand cDNA was prepared using the Superscript First Strand Kit (Invitrogen, Carlsbad, CA, USA; ref: 11904-018). Quantitative real-time PCR was performed using LightCycler 480 SYBR Green I master mix (Roche Applied Science, ref: 04887352001) and analyzed on a LightCycler 480 Instrument II system (Roche Applied Science), according to the manufacturer’s instructions. The comparative method of relative quantification (2^−ΔΔ*CT*^) was applied to calculate the expression levels of each target gene, which were then normalized for dog GAPDH mRNA. Table 1 lists the primers used in this study.

**Table 1.** List and Sequences of Primers Used for Quantitative RT-PCR.

Gene	Forward primer (5′ to 3′)	Reverse primer (5′ to 3′)	Product size
GAPDH	TCGCCATCAATGACCCCTTC	TTCCCGTTCTCAGCCTTGAC	106
Insulin receptor (INSR)	GCACGTATGGAGCCAAGAGT	AGTGCGTGATATTGCCATTGG	153
Insulin	GGCTCTGTACCTGGTGTGC	CACTGCTCCACGATGCCTC	174
Glucagon receptor (GLP1 R)	CCGGGCTCCTTTGTGAATGT	AGGGCAAGCTGGAGTTGTG	136
Glucagon	CCAGGATTTCGTGCAGTGGT	GCAATGAATTCCTTGGCAGCT	150
Somatostatin	CATCGTCCTGGCTCTGGG	TGGTTGGGTTCAGACAGCAG	144
Amylase	AGACATGGTGACTCGGTGTAAC	TGGGACCGCTGGAAAATCTC	156
PDX1	GCTGCCTTTCCCGTGGAT	AGTCCGTTTGTTTTCTTCTGGC	100

### Insulin Secretion

Insulin secretion was studied only with the PIs derived from neonatal pancreases. PIs (2×10^5^) were introduced into Millicell^®^ cell culture inserts (Merck Millipore, Burlington, MA, USA) and incubated overnight in 24-well plates in culture medium containing 2.8 mM glucose then for 60 min in HEPES-buffered Krebs-Ringer Buffer (KRB) (115 mmol/L NaCl, 5 mmol/L KCl, 1 mmol/L CaCl_2_, 1 mmol/L MgCl_2_, 24 mmol/L NaHCO_3_, 10 mmol/L HEPES pH 7.4, and 0.2% bovine serum albumin) containing 2.8 mM glucose. Stimulated insulin secretion was then measured by static incubation for 60 min in KRB containing either 2.8 mM or 15 mM glucose. For insulin content measurement, cells were lysed directly in the culture wells with TETG solution (20 mM Tris pH 8.0; 0.1% Triton X-100; 1% glycerol; 137 mM NaCl; 2 mM EGTA) and protease inhibitor tablet (Roche Applied Science) for 5 min on ice. The lysate was centrifuged at 3000 rpm for 5 min and stored at –20°C until insulin measurement. The insulin values in the supernatant were expressed as a percent of insulin content in the PIs seeded for each experiment. Insulin secretion and intracellular content were measured in duplicate by ELISA.

### PI Transplantation

#### Animals

PIs were transplanted into male SCID mice aged 6–8 weeks (CB-17/Icr-Prkdc scid/Rj, Janvier Labs, Le Genest-Saint-Isle, France). When indicated, diabetes was induced by streptozotocin (Sigma-Aldrich, Saint Louis, MI, USA) freshly prepared in citrate buffer and injected intraperitoneally to the mice in a dosage of 200 mg/kg body weight. Blood samples were collected from the tail at regular intervals over the next 2 days and used to measure glucose levels with glucose strips (Accu-Chek, Roche, France) and a glucose meter. For the experiment, we selected mice whose blood glucose 1–2 days after the streptozotocin injection was above 2.5 mg/L.

#### PI transplantation into SCID mice

To assess PI transplant survival in SCID mice, 1 million PIs prepared from F55 specimens were grafted below the kidney capsule of four mice as described previously^16^. Blood was collected for serum insulin measurement before sacrifice 2 (*n* = 2), 4 (*n* = 2), or 6 (*n* = 5) months after transplantation. The transplant was dissected and prepared for morphological examination and immunohistochemistry localization of alpha and beta cells.

#### PI transplantation into diabetic SCID mice

To evaluate the in vivo function of canine PIs prepared as described above, 15 SCID mice were given a streptozotocin injection. Among them, 12 had blood glucose levels above 2.5 g/L and were investigated further. To maximize survival, a Linbit slow-release insulin capsule (LinShin, Scarborough, Ontario, Canada) designed for mice and lasting 3–4 weeks was implanted subcutaneously. In six mice, a transplant of 10^6^ PIs was grafted below the kidney capsule as described previously^16^. The other six mice served as controls. Blood glucose concentrations were measured at regular intervals in the morning after a 4-h fast. After 60 days, nephrectomy was performed in grafted mice to remove the transplants and assess their contribution to blood glucose control. The mice were sacrificed 24 h later. Each transplant was dissected from the adjacent kidney and studied by immunohistochemistry. In two mice from the transplanted and control groups, the pancreases were also dissected to assess beta-cell destruction by streptozotocin.

### Insulin Assay

Insulin was measured in duplicate in the media, cell extracts, and serum of SCID mice (see below) using a canine insulin ELISA (Mercodia, Uppsala, Sweden).The antibody used in this assay does not cross-react with mouse insulin. By using this ELISA kit we can therefore detect and quantify dog insulin in serum from transplanted SCID mice as the method will not recognize and measure mouse insulin.

### Statistics

Statistical analyses were conducted using two-tailed Mann–Whitney *U*-tests (GraphPad Prism 6) to compare: (i) differences in insulin secretion following glucose stimulation; and (ii) blood glucose in mice transplanted or not with PIs at all different time points.

## Results

### Production of PIs

**Figure 1.**
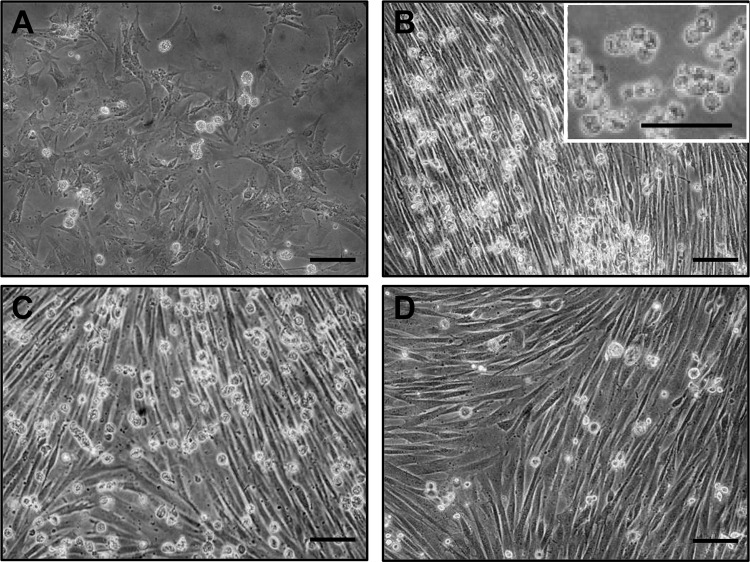
Fetal pseudoislets (PIs) produced, during a period of 2 weeks in culture, by collagenase-digested pancreatic tissue from six 53-day-old dog fetuses. Sequence of morphologic events 3 (A), 6 (B), 9 (C), and 13 (D) days after culture initiation. Fibroblast-like cells proliferated, coating the bottom of the culture dish. PIs as well as pancreatic cell clusters were first detected on day 3. On day 6, numerous free-floating PIs were visible. The number of PIs continued to increase subsequently. After 2 weeks, PI production decreased to nothing (not shown). The inset in Figure 1B is a photomicroscopic view of the culture dish showing free-floating PIs. Scale bars 100 µm.

A characteristic sequence of events occurred when the fetal or postnatal pancreas specimens treated as described above were seeded on Petri dishes. Tissue fragments were visible during the first few days then disappeared gradually. After 3–4 days, abundant fibroblasts coated the bottom of the dish. As shown in Fig. 1, round structures that were either attached to the bottom of the dish or free-floating appeared gradually. PIs were first detected on day 3 then increased in number to a peak 7–10 days after seeding. From then on, a greater number of PIs detached from the fibroblast monolayer to float freely in the culture medium. After 3 weeks, PI production decreased gradually to nothing. As shown in the Fig. 1B inset, the PIs were isolated and well identified at first but then rapidly formed aggregates, making it difficult to measure exact PI diameters and PI counts. The number of PIs produced was nonetheless estimated at about 3×10^5^ per litter for F45 specimens, 10^7^ L for F55 specimens, and from pancreas obtained at D1, and W3. The tissue fragment from adult pancreas produced fewer than 10^5^ PIs.

### Tissue Characterization by Immunohistochemistry

**Figure 2.**
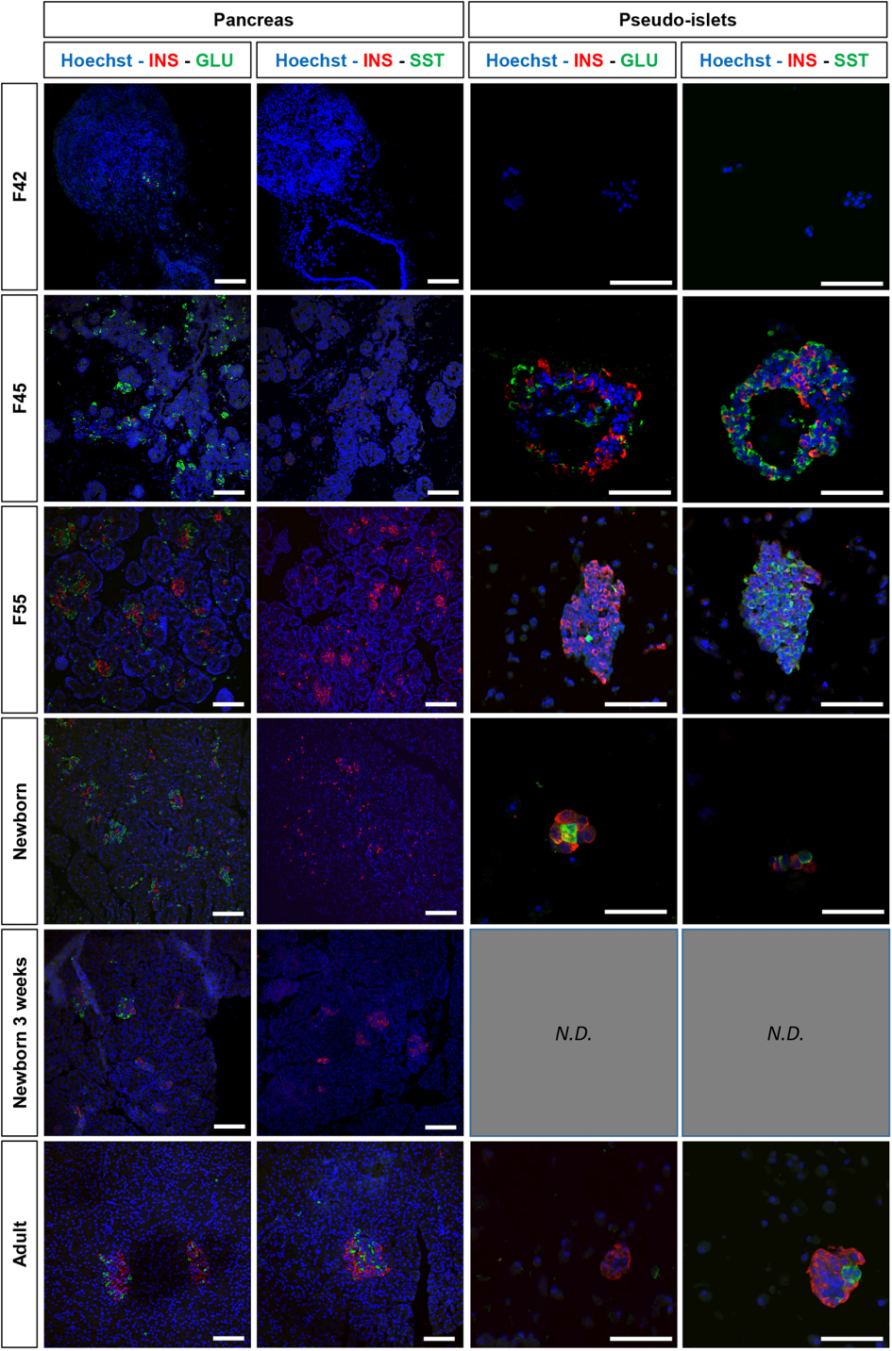
Light micrograph of pancreatic tissue from dogs at various ages and of the pseudoislets (PI) they produced after collagenase digestion. The sections were immunostained for insulin, glucagon, and somatostatin. The figure shows 4-μm paraffin sections of pancreases and derived PIs at fetal days 42, 45 and 55; postnatal days 1, 21; and adulthood. Lanes A and B show endocrine-cell distribution within the gland and lanes C and D endocrine-cell distribution within the corresponding PIs cultured for 9 days. Insulin (red) and glucagon (green) stains are merged in lanes A and C. Insulin (red) and somatostatin (green) stains are merged in lanes B and D. The nuclei are stained in blue with Hoechst. At fetal days 45 and 55, large aggregates of PIs composed of endocrine-cell clusters are visible. Scale bars: 50 μm. Note that, in the pancreas, insulin cells appear at the late fetal stage and islet structures during the postnatal period. Somatostatin-positive cells were not seen before birth. In contrast, PIs exhibited rapid maturation with the appearance of insulin- and somatostatin-positive cells after a few days in culture.

Fig. 2 illustrates islet formation and endocrine-cell development in the pancreas. Consistent with our previous data^16^, from F42 to F45, alpha cells were abundant and beta cells scarce. Aggregates of alpha and beta cells were seen at F55 and fully formed islets 3 weeks after birth and in the adult pancreas. Surprisingly, somatostatin-containing cells (delta cells) were seen only in the adult pancreas.

PI production from digested pancreatic tissue is shown in the vertical lanes 3 and 4 of Fig. 2. With the F42 tissue, no PIs were produced and the cell pellets contained no endocrine cells. In contrast, tissues from later stages (F45, F55, and D1) produced PIs that formed large aggregates and contained numerous alpha and beta cells. By contrast to findings in the intact pancreas, delta cells were detected at all the developmental stages studied. PIs were produced in smaller numbers by the adult tissue, in which the endocrine-cell distribution was similar to that in the neonatal period, with abundant insulin-positive cells, presence of alpha cells, and numerous delta cells.

### Pancreatic Marker mRNA Quantification in PIs from Pancreases at Different Developmental Stages

**Figure 3.**
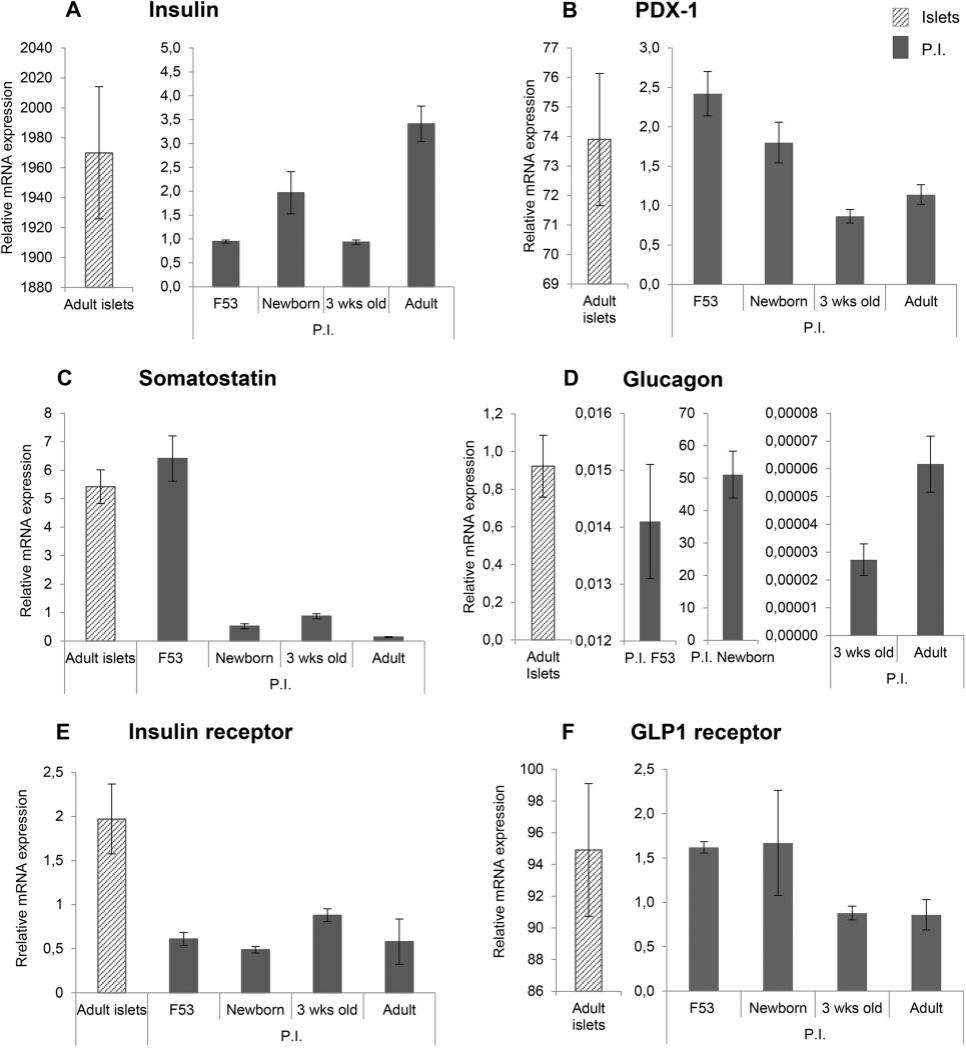
Quantitative real-time PCR (qRT-PCR) assessment of beta-cell markers. Expression of the beta-cell markers insulin, glucagon, and somatostatin; PDX-1; insulin receptor and GLP1 receptor was evaluated in PIs from pancreatic tissues at various developmental stages, using qRT-QPCR. The results were compared to the corresponding values found in pancreatic islets from an adult dog. Expression is normalized for GAPDH. The results are reported as mean ± S.E.M. from three independent mRNA preparations.

As reported above, PI production efficiency and PI cell composition varied across developmental stages. To further characterize the PIs generated at each stage, we studied the mRNA expression of various pancreatic markers, using qRT-PCR. Changes in the mature endocrine component of the PIs were monitored using insulin, glucagon, and somatostatin staining. The transcription factor Pdx1 was quantified to monitor both endocrine progenitor cells and insulin-positive cells. We also assessed the expression of insulin- and GLP1-receptors. We compared the expression level of each marker in PIs at the different developmental stages and in the adult pancreatic islets. Insulin expression was greatest in PIs from newborn and adult pancreatic tissue, where it was nonetheless more than a thousand times lower than in adult islets (Fig. 3). Glucagon expression was also strongest in newborn PIs, where it was considerably higher than in adult islets. Somatostatin expression was highest in F53 PIs, where it was similar to that in adult islets. The low level of insulin expression in PIs compared with adult islets suggested that the main cells expressing Pdx1 were pancreatic progenitors. Therefore, the decrease in Pdx1 expression by PIs over time was probably related to the concomitant decrease in the number of pancreatic progenitors in the pancreatic tissue. Finally, insulin and GLP1 receptor expression in PIs remained unchanged over time and similar to that seen in adult islets.

### Beta-Cell Function

We first evaluated beta-cell function by measuring the insulin content of PIs produced by the two D1 pancreatic specimens. The insulin content was 24 and 35 pg/PI, respectively.

**Figure 4.**
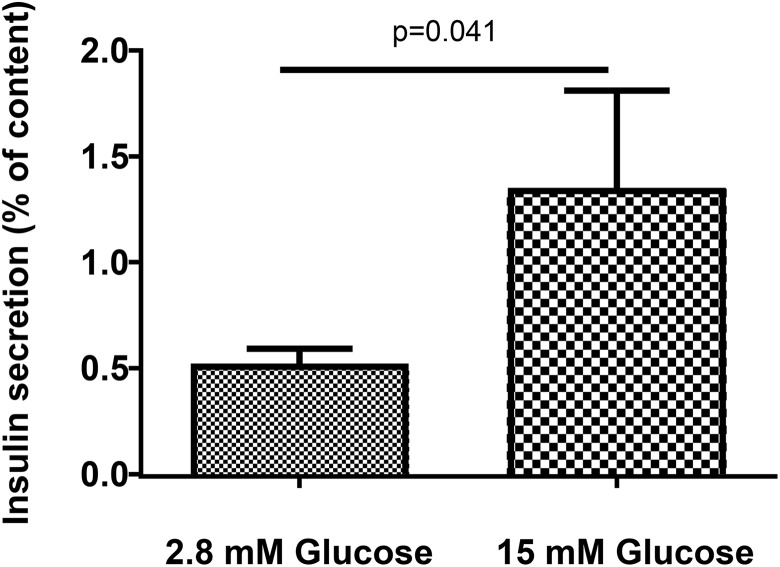
Glucose stimulation of insulin secretion by pseudoislets (PIs) in vitro during static incubation. Insulin release by six different preparations of PIs derived from two 1-day-old pups. The PIs were incubated in medium containing 2.8 mM glucose for 1 hour then incubated for another hour in medium containing 2.8 or 15 mM glucose. Each static incubation was performed in triplicate. Insulin was measured in duplicate in the supernatant and PIs. Insulin production is expressed as a % of the PI insulin content. The data are the means of six experiments. Two-tailed Mann–Whitney *U*-test used to assess whether differences in insulin secretion according to glucose concentration was significant (*p*-values are shown). The stimulation index defined as the ratio of stimulated insulin with 15 vs. 2.8 mM glucose was 3.

Then, we measured in vitro insulin secretion by PIs from the same D1 specimens during static incubation in medium containing 15 mM or 2.8 mM glucose. With 15 mM glucose, insulin secretion was stimulated (Fig. 4). The stimulation index defined as the ratio of insulin secreted with 15 mM over 2.8 mM glucose was 3. These findings established that D1 PIs released insulin into the medium in a manner that was sensitive to the glucose concentration.

### In Vivo Survival of Transplanted PIs

**Figure 5.**
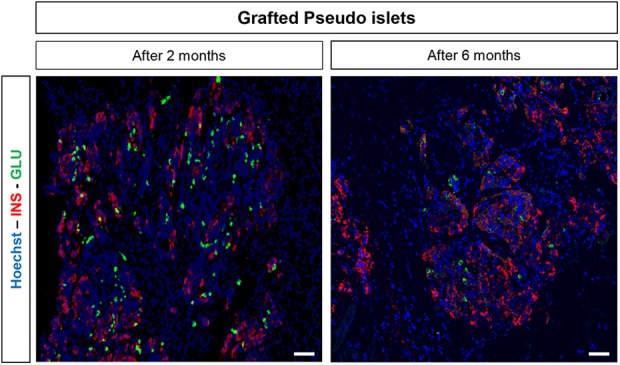
Survival and differentiation of pseudoislets (PIs) after transplantation into SCID mice. PIs from 52-day-old fetuses were harvested and transplanted under the kidney capsule of two non-diabetic SCID mice. The transplants were removed 2 or 6 months later and prepared for immunostaining of insulin (red) and glucagon (green). Nuclei seen by confocal microscopy were stained in blue with Hoechst. Note the transplant maturation between 2 and 6 months, with an increase in insulin-positive cells and a decrease in glucagon-positive cells. Scale bar: 50 µm.

Survival of PIs produced from F55 fetal pancreas and grafted into SCID mice was assessed 2 and 6 months after transplantation as illustrated in Fig. 5, A and B. The transplant was easily identified and dissected. Its overall endocrine-cell distribution was similar to that of PIs in 1-week-old cultures (Fig. 2). PIs transplanted for 2 months contained large numbers of alpha cells. The 6-month-old transplants contained fewer alpha cells, contrasting with a far greater number of beta cells. Serum insulin levels obtained before sacrifice of the six mice transplanted for 6 months ranged from 6 mU/L to 16.7 mU/L. The serum insulin level in the transplanted mouse shown in Fig. 5B was 6.4 mU/L. No dog insulin was detected in serum samples from control non-transplanted mice.

### PI Transplantation into Diabetic SCID Mice

**Figure 6.**
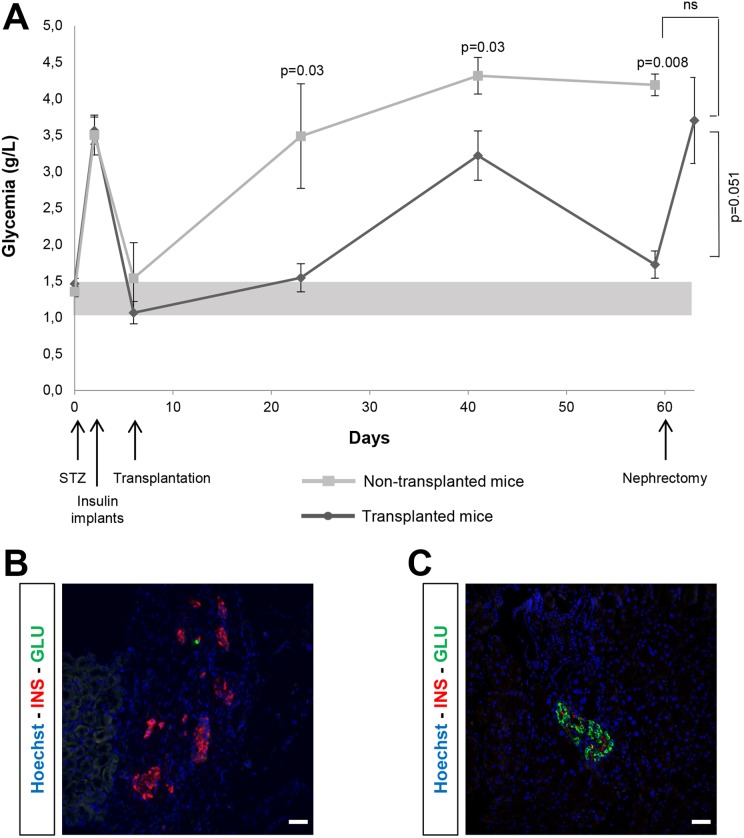
Blood glucose effects of pseudoislet (PI) transplantation into diabetic SCID mice. (A): In 12 SCID mice, diabetes was induced by an intraperitoneal streptozotocin (STZ) injection. Implants releasing insulin for 2–4 weeks were inserted 3 days later. At the end of the first week, 10^6^ PIs were transplanted under the kidney capsule of six mice. The other six mice served as controls. Each point is the mean ±S.E.M. of blood glucose levels measured at regular intervals in both groups of mice. Two-tailed Mann–Whitney *U*-test was used to assess whether differences in blood glucose between the two groups of mice was significant (*p*-values are shown). After 2 months, the graft was removed by nephrectomy, and blood glucose was measured on the following day. (B): Insulin (red) and glucagon (green) staining of the graft removed after 2 months. The transplanted tissue is clearly visible, with multiple insulin- and scarce glucagon-containing cells. Scale bar: 50 µm. (C): The pancreases of transplanted mice and controls were dissected and prepared for immunochemistry. An islet containing glucagon-positive cells but virtually no insulin-positive cells is shown. Scale bar: 50 µm.

After streptozotocin injection, mean blood glucose ranged from 3.5 to 4 g/L (Fig. 6A). Subcutaneous injection of slow-release insulin capsules rapidly returned the blood glucose levels to normal. The control group (no PI transplantation) had severe diabetes with blood glucose levels above 3g/L. PI transplantation significantly decreased blood glucose levels compared with controls but returned them to the normal range in only two of the six animals. PI transplant removal was followed by a significant increase in blood glucose levels to the values seen in the controls. Immunohistochemistry staining for insulin and glucagon of a PI transplant removed after 2 months showed numerous beta cells and, surprisingly, very few alpha cells (Fig. 6B). In Fig. 6C, the almost complete absence of beta cells illustrates the severity of the diabetes induced by streptozotocin injection.

## Discussion

Although the islet isolation procedure has been improved over time, it still involves pancreatic tissue digestion by collagenase (usually injected through the Wirsung canal), followed by manual or mechanical islet separation. In human and most animal species this procedure is not feasible in the perinatal period, chiefly because the Wirsung canal is too small or the islets incompletely formed or damaged by the collagenase. The report by Hellerström et al.^1^ that fetal pancreatic PIs can be isolated from cultures of pancreatic tissue previously subjected to mild collagenase digestion prompted several groups to investigate PI production from neonatal pancreases from various species^2–7,17,18^.

The data reported here indicate that PIs can be produced ex vivo from canine fetal or postnatal pancreatic tissue, thus adding the dog to the species available for studying PI development and function. In our experience, PIs cannot be obtained from canine pancreatic tissue at early fetal stages. In contrast, we obtained PIs from pancreatic tissue specimens harvested during the last quarter of gestation, i.e., from F45 onward, after birth, and in adulthood. PI production efficiency varied according to donor age, being lowest at F45 and increasing thereafter. Adult pancreatic tissue also produced PIs, albeit less efficiently than tissue at earlier stages. The exact yield of PIs production from the pancreas cannot be determined as only a small fragment of the total gland was used in our experiment. The exact number of PIs could not be determined because, unfortunately, the fragment dissected from the whole gland could not be weighed under sterile conditions. More importantly, the distribution and morphology of beta cells, and probably also of precursor cells, are extremely heterogeneous in the canine pancreas^19–21^. Numerous fragments taken from the left to the right lobe of the gland should therefore be examined to evaluate the efficiency of PI production by the entire gland. We cannot totally exclude that some PIs obtained by tissue culture come from preexisting islets. However, changing the medium every 3 days eliminated any floating undigested pancreas fragments, which were not seen after day 3 of culture. More importantly, the comparison of endocrine-cell compositions in the intact pancreas before collagenase digestion and in the PIs obtained from these glands clearly showed evidence of maturation during the tissue culture process. We have reported previously^16^ that canine fetal beta cells appear in the pancreas at F40, that alpha cells predominate during fetal life, and that fully formed islets are observed in neonates but not in fetuses. The additional data reported here indicate that somatostatin-expressing delta cells emerge very late during development, as they were seen only in the adult pancreas. At all developmental stages, PIs differed markedly from intact pancreases regarding cell composition, with a predominance of beta cells, scarce alpha cells, and, importantly, the presence of delta cells. Such variation of cell type composition could be due to various mechanisms. It has been shown that rat islets transplanted for 12 weeks have lost a large percentage of non-beta cells^22^. The high prevalence of beta cells observed in the PIs in our study could be due to the differential survival of beta cells in culture and the death of alpha cells. However we believe that in this study (obviously in a different model) the presence of numerous delta cells indicates a real maturation process.

During pancreatic morphogenesis, the transcription factor Pdx1 appeared in the pancreatic progenitor cells and was subsequently expressed by mature beta cells^23^. Expression of Pdx1 mRNA was strong in PIs from F55 specimens and decreased thereafter. Although this study was not designed to elucidate the mechanism of endocrine-cell development, the cell composition differences between intact pancreases and PIs suggest that the endocrine cells were derived from pancreatic progenitors.

The observation that PIs transplanted into non-diabetic SCID mice survived is also an important finding. Although the volume of the transplants after 6 months was not measured, it was clearly greater than at transplantation, and the transplants received an abundant blood supply as shown by the number of capillaries at the surface of the transplanted tissue. Furthermore, during 2–6 months after transplantation, alpha-cell and delta-cell numbers decreased relative to beta-cell numbers. This finding suggests that beta cells were newly formed in the transplants from undifferentiated progenitors expressing Pdx1. Qualitative and quantitative evaluations of the expression of neurogenin-3 (Ngn3), the transcription factor that controls endocrine commitment in the developing pancreas, would have been of interest. Unfortunately our Ngn3 antiserum did not recognize the protein in dog pancreas, even during early gestation. The sequence of dog Ngn3 has not been fully established, and consequently efficient quantitative RT-PCR probes for Ngn3 cannot be designed.

The insulin content measured in two PI samples from D1 specimens was far lower than values reported in adult dog pancreatic islets. In a recent study of adult dogs, the insulin content was 0.34 µg/islet^23^, i.e., 10^4^-fold the content in these PIs. In addition, the qRT-PCR results showed far lower insulin mRNA expression in PIs than in adult islets. The PIs stimulation index was 3, compared with 4–6 in studies of adult canine islets^24–26^, although differences in the techniques used obscure the comparison. Overall, our findings are consistent with those of previous studies of fetal or neonatal PIs from rats, pigs, sheep, and humans showing a poor insulin response of PIs to glucose.

Canine PIs transplanted into non-diabetic SCID mice survived, grew, developed large numbers of beta cells, and released insulin into the bloodstream. In diabetic mice, the insulin released from the transplants lowered the blood glucose levels, although these returned to normal value in only two of six animals. After transplant removal from the diabetic animals, the blood glucose levels increased to their pre-transplant values. Several hypotheses can be put forward to explain the incomplete blood glucose control in the transplanted diabetic mice. First, the number of transplanted beta cells may have been insufficient. In a study of neonatal porcine islets obtained using a similar method and transplanted into diabetic mice^5^, a higher number of insulin-positive cells in the transplant was associated with better glycemic control. Second, the transplantation period may have been too short to allow sufficient beta-cell development. In the above mentioned study^5^, the insulin content increased 20- to 30-fold during the transplantation period. In our protocol, the PIs were implanted 8 weeks and a longer period of implantation might be necessary to see a clearer effect of the PIs implants on metabolic control.

Our results demonstrate that neonatal dog pancreatic tissue can serve as a source of PIs capable of releasing insulin both in vitro and in vivo. When transplanted into SCID mice, these PIs survive and release insulin into the host bloodstream. Furthermore, when transplanted into diabetic SCID mice, these PIs achieved some degree of hyperglycemia control, although most animals failed to achieve euglycemia.

Spontaneous diabetes has been reported in several animal species^27^. The dog is the second most often used animal species for studies of diabetes, after rodents. The similarities between human type 1 diabetes and canine diabetes contribute to this interest^28–30^.

In conclusion, neonatal canine pancreatic tissue can be used to produce large numbers of functional beta cells that remain viable when transplanted into immune-incompetent mice. As described this method has some important limitations for application in veterinary medicine. The material used has been collected for research purpose in a university veterinary hospital. Collecting a larger amount of pancreas will raise important ethical issues which must be overcome. To be acceptable, in the future canine pancreas will be procured from dogs euthanized for reasons which do not interfere with the pancreatic function, and with the owner’s consent. The second limitation is the low yield of PIs production. Developing innovative techniques to expand the cells after PIs isolation would solve this difficult issue
